# Influencing Factors to mHealth Uptake With Indigenous Populations: Qualitative Systematic Review

**DOI:** 10.2196/45162

**Published:** 2023-06-23

**Authors:** Andrew Goodman, Ray Mahoney, Geoffrey Spurling, Sheleigh Lawler

**Affiliations:** 1 School of Public Health The University of Queensland Turrbal, Jagera Country, Herston Australia; 2 Australian eHealth Research Centre (AEHRC) Commonwealth Scientific and Industrial Research Organisation (CSIRO) Turrbal, Jagera Country, Herston Australia; 3 College of Medicine and Public Health Flinders University Kaurna Country, Adelaide Australia; 4 General Practice Clinical Unit The University of Queensland Turrbal, Jagera Country, Herston Australia

**Keywords:** mHealth, Indigenous, Canada, Australia, New Zealand, United States, Papua New Guinea, Samoa, qualitative, systematic review, feasibility, acceptability, users, design, workflow

## Abstract

**Background:**

The advancements and abundance of mobile phones and portable health devices have created an opportunity to use mobile health (mHealth) for population health systems. There is increasing evidence for the feasibility and acceptance of mHealth with Indigenous populations. Providing a synthesis of qualitative findings of mHealth with Indigenous populations will gain insights into the strengths and challenges to mHealth use in Indigenous populations.

**Objective:**

This review aimed to identify and synthesize qualitative data pertaining to the experiences and perceptions of mHealth from the perspectives of end users (patients and service providers) living in the colonial settler democracies of Canada, Australia, New Zealand, the United States, the Pacific Islands, and the Sápmi region of northern Europe.

**Methods:**

In May 2021, systematic searches of peer-reviewed, scientific papers were conducted across the 5 databases of PubMed, CINAHL, Embase, PsycINFO, and Web of Science. Qualitative or mixed method studies were included where a mHealth intervention was the primary focus for responding to health challenges with Indigenous populations. Two authors independently screened papers for eligibility and assessed the risk of bias using a modified version of the Critical Appraisal Skills Programme. A meta-aggregative approach was used to analyze the findings of included studies.

**Results:**

Seventeen papers met the eligibility criteria, 8 studies with patients, 7 studies with service providers, and 2 studies that included both patients and service providers. Studies were conducted in Australia (n=10), Canada (n=2), New Zealand (n=2), Papua New Guinea (n=1), the United States (n=1), and Samoa (n=1). Our interpretation of these qualitative findings shows commonalities between Indigenous patients’ and service providers’ perceptions of mHealth. We summarize our findings in six themes: (1) mHealth literacy, (2) mHealth as a facilitator for connection and support, (3) mHealth content needed to be culturally relevant, (4) mHealth security and confidentiality, (5) mHealth supporting rather than replacing service providers, and (6) workplace and organizational capacity.

**Conclusions:**

This research suggests that mHealth can meet the needs of both patients and service providers when the mHealth intervention is culturally relevant, accounts for digital and health literacy, incorporates interactive components, is supported by workplaces, fits into health provider workflows, and meets security and confidentiality standards. Future mHealth research with Indigenous populations should partner with key representatives (eg, patients, service providers, and executive leaders) in the mHealth design appropriate to the purpose, people, setting, and delivery.

## Introduction

The technological advancements and abundance of mobile phones and portable health devices have created a plethora of mobile health (mHealth) tools. mHealth is defined as “the use of mobile devices—such as mobile phones, patient monitoring devices, personal digital assistants and wireless devices—for medical and public health practice” [1]. These include mobile phone apps, text messages, portable monitoring devices and electronic patient information.

Systematic reviews globally have suggested mHealth is a broadly feasible and effective resource for a range of health conditions including; behavior change [2,3], noncommunicable disease management [4-9], perinatal care [10,11] medication adherence [12], and mental health well-being [13,14]. Likewise, health care workers suggest mHealth improves patient health outcomes and increases peer communication and care coordination [15,16].

There is a growing number of qualitative studies exploring the views and perceptions of mHealth from 2005 onward, resulting in a number of qualitative systematic reviews [16-21]. Findings from these reviews provide a collective insight into user perceptions and experience of mHealth to influence future research and implementation. These systematic reviews predominantly focus on non-Indigenous populations and fail to explore the user experiences of Indigenous people and their service providers. We need to ensure a space is kept privileging Indigenous worldviews as it pertains to mHealth. mHealth interventions are being explored with Indigenous populations with increasing interest [22-24]. Reviews examining the applicability of mHealth for Indigenous populations exist, and these indicate it is an acceptable health resource [23,24]. Yet, these reviews include qualitative data as only a peripheral focus and are inconsistent with the intervention type [23], and outcomes [24].

Providing a synthesis of qualitative findings of mHealth with Indigenous populations will gain insights to the strengths and challenges to mHealth use in Indigenous populations. This review aimed to identify and synthesize qualitative data pertaining to the experiences and perceptions of mHealth with Indigenous populations and the service providers that work with Indigenous populations.

## Methods

### Overview

A systematic search was conducted of peer-reviewed literature for this qualitative synthesis. A protocol of this qualitative synthesis was registered with the International Prospective Register of Systematic Reviews (PROSPERO; registration number CRD42021251861). We extracted qualitative data pertaining to the experiences and perceptions of both patients (Indigenous peoples) and service providers (either Indigenous or non-Indigenous health policy makers, health care professionals, and researchers) who work with Indigenous peoples from Canada, Australia, New Zealand, the United States, the Pacific Islands, and the Sápmi region of northern Europe. We define Indigenous Peoples as “distinct social and cultural groups that share collective ancestral ties to the lands and natural resources where they live, occupy or from which they have been displaced” [25].

### Search Strategy and Selection Criteria

A comprehensive list of search terms and strings were developed with the assistance of a librarian with expertise in systematic reviews. Systematic searches of peer-reviewed, scientific papers in English were conducted across 5 databases in May 2021: PubMed, CINAHL, Embase, PsycINFO, and Web of Science. Qualitative or mixed method studies were included where a mHealth intervention was the primary focus for responding to health challenges with Indigenous populations. As such, experimental and quasi-experimental studies were considered, as long as they met the following inclusion criteria:

- Participants: Indigenous people of all ages from Canada, Australia, New Zealand, United States, the Pacific Islands, the Sápmi region of northern Europe; OR are service providers (either Indigenous or non-Indigenous) who work with Indigenous persons from Canada, Australia, New Zealand, the United States, the Pacific Islands, the Sápmi region of northern Europe; OR where participants are multicultural, outcomes for Indigenous persons are reported specifically.
- Interventions: primary focus was a mHealth intervention delivered using a wireless device (eg, mobile or tablet app, website designed for mobile, messaging [SMS, voice, multimedia messaging system, etc]). The mHealth intervention aims to address a health challenge (eg, diagnosis of disease, substance use, health behaviors, quality of life, health knowledge, self-efficacy, caregiver support, etc).
- Outcomes: studies reported on one or more outcomes including user; experiences, perceptions, barriers, and enablers via qualitative research methods (eg, interviews and focus groups).

A sample of the search strings using text words and subject heading keywords for PubMed can be found in Multimedia Appendix 1. The use of proximity operators, truncation, and phrase searching was used to widen the search to capture all iterations of both the mHealth and Indigenous themes. The 2 search strings were then combined to narrow the results—enabling discovery of all possible scientific papers, which capture mHealth interventions with Indigenous populations from Canada, Australia, New Zealand, the United States, the Pacific Islands, and the Sápmi region of northern Europe. The qualitative papers were then identified via screening by 2 researchers (AG and SL).

### Data Extraction and Quality Appraisal

Initial database searches and duplicate removal were conducted by 1 author (AG). Screening, review, and extraction were assisted by the web-based systematic review program Covidence (Veritas Health Innovation) [26]. Two authors (AG and SL) independently screened titles and abstracts against the inclusion criteria, and papers clearly not meeting the inclusion criteria were excluded.

Subsequently, 2 authors (AG and SL) screened the full-text papers independently and then discussed for comparison. Any differing views were resolved through discussion. Manual searches of reference lists were conducted on full-text papers included in the review. A final list of full-text papers and their citations which met inclusion criteria were downloaded and saved using Covidence software.

The quality of the included studies was appraised using a modified version of the Critical Appraisal Skills Programme (CASP) qualitative checklist [27]. An additional question from the Joanna Briggs Institute (JBI) was added that related to locating the researchers cultural or theoretical standpoint [28], improving the cultural rigor of this critical appraisal tool.

### Data Analysis and Synthesis

The data included in the analysis were all text included in the “Results” or “Findings” sections of the papers (excluding purely quantitative findings) and was extracted from the papers into NVivo 12 Plus software (QSR International) [29]. Characteristics of each study to be extracted for descriptive purposes included: Indigenous identification, study location (country), year, sample size, participant demographic characteristics (age, gender), data collection, and analysis methods.

A meta-aggregative approach was used to analyze the findings [30]. This analysis approach privileges the findings, presented as “themes” or “constructs” in qualitative research, as identified by the researchers (not the reviewer). This method helps ensure the expanse and breadth of cultural learnings identified by researchers conducting the original studies are not lost by the reviewers.

## Results

### Overview

**Figure 1:**
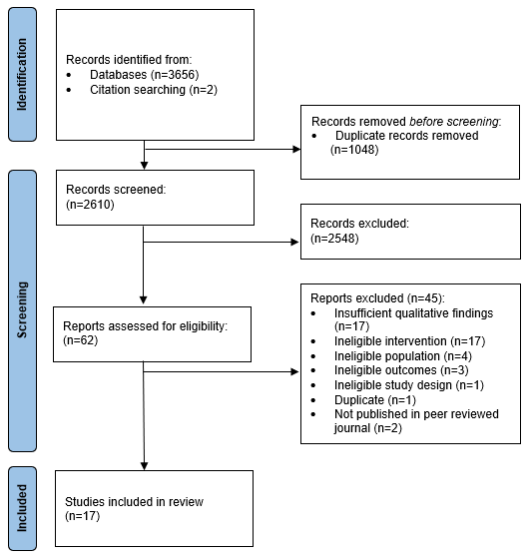
Preferred reporting items for systematic reviews and meta-analyses flow diagram of study selection.

From database searches, 2608 unique papers were identified; 2 additional papers were located by manual searches. In total, 2610 titles and abstracts were reviewed against the inclusion criteria, of which 2548 were excluded, leaving 62 papers for full-text review. Following the full-text review, 45 papers were excluded, leaving 17 studies included in this qualitative systematic review (Figure 1).

### Description of Included Studies

All 17 studies included in this review were published between 2013 and 2021. Eight were studies specifically with Indigenous patients [31-38]. Seven studies were with service providers (either Indigenous or non-Indigenous) who work with Indigenous peoples [39-45]. Two studies involved both patients and service providers in data collection [46,47], so findings were considered for both.

Characteristics of the 17 studies are shown in Table 1. Ten papers involved Aboriginal and Torres Strait Islander peoples of Australia [31,36-38,40,42,44-47], 2 with the First Nations, Inuit, or Métis peoples of Canada [34,39], 2 with the Māori peoples of Aotearoa, New Zealand [33,35], 1 paper with the Indigenous peoples of Papua New Guinea [43], 1 paper with the Native Hawaiian and Pacific Islander peoples of Hawaii, the United States [41], and 1 paper with the Indigenous peoples of Samoa [48]. We were unable to identify any papers with Indigenous people of the Sápmi region of northern Europe that met the review criteria.

**Table 1:** Characteristics of included studies.

Studies		Indigenous peoples (country)	Focus area of intervention	Type of mHealth^a^ delivery	Participants (roles)	Method	CAT^b^ [27,28]
**Indigenous patients**							
	Kennedy et al [38]	Aboriginal and Torres Strait Islander (Australia)	Perinatal health care	App^c^	8 Indigenous patients	Interviews^d^	10
	Tighe et al [37]	Aboriginal and Torres Strait Islander (Australia)	Suicide prevention	App^e^	13 Indigenous patients	Interviews^d,f^	8
	Jongbloed et al [34]	First Nations, Inuit, and Métis (Canada)	Illicit drug use	mHealth broad concept	130 Indigenous patients	Questionnaire^g^	6
	Peiris et al [36]	Aboriginal and Torres Strait Islander (Australia)	Smoking cessation	App^e^	15 Indigenous patients	Interviews^h^	8
	Gasteiger et al [35]	Māori (New Zealand)	Pregnancy or perinatal health care	mHealth broad concept	Nine Indigenous patients	Interviews^d,f,i^	11
	Te Morenga et al [33]	Māori (New Zealand)	Healthy lifestyle	mHealth broad concept	21 Indigenous patients	Focus group and “bus stop activity”^d,i^	9
	McCool et al [48]	Samoa	Smoking cessation	Text message	36 Indigenous patients	Focus group^d,f^	9
	Povey et al [31]	Aboriginal and Torres Strait Islander (Australia)	Mental health well-being and suicide prevention	App^e^	9 Indigenous patients	Focus group^h^	10
**Service providers**							
	Akearok et al [39]	Inuit (Canada)	Social determinants view of health	App^e^	5 (health service recruitment and education staff)	Interviews and survey^j^	3
	Raphiphatthana et al [42]	Aboriginal and Torres Strait Islander (Australia)	Mental health well-being	App^e^	57 (nurses, support workers, Indigenous health workers, psychologists, and alcohol and other drug workers)	Interviews^d,h^	10
	Macniven et al [45]	Aboriginal and Torres Strait Islander (Australia)	Cardiovascular	ECG attached to a mobile phone (iECG)	18 (Indigenous health workers, registered nurses)	Interviews^d,f^	10
	Bennett-Levy et al [44]	Aboriginal and Torres Strait Islander (Australia)	Mental health well-being	App^e^	28 (consultant trainers, youth workers, Indigenous service workers, drug and alcohol worker, family development worker, well-being coordinator, Aboriginal health education officer, mental health support worker, and healthy lifestyle worker)	Interviews and field notes^d^	9
	Yazdanshenas et al [41]	Native Hawaiian and Pacific Islander (United States)	Hypertension	Text message	20 (executive leader, church leader, community advocate, and health care providers)	Interviews^h^	7
	Dingwall et al [40]	Aboriginal and Torres Strait Islander (Australia)	Mental health well-being	App^e^	15 (health professionals, managers, program coordinators, and an Aboriginal elder)	Interviews^d^	10
	Kurumop et al [43]	Papua New Guinea	Malaria	SMS Text message	17 (health workers)	Focus group^h^	9
**Both Indigenous patients and service providers**							
	Brown et al [46]	Aboriginal and Torres Strait Islander (Australia)	Mental health well-being	mHealth broad concept	12 (8 Indigenous health workers, 4 Indigenous patients).	Focus group^h,k^	11
	Houston et al [47]	Aboriginal and Torres Strait Islander (Australia)	Perinatal health care or parenting	App^e^ and website	31 (21 administration staff, pediatricians, child health nurses, general practitioners, and Indigenous health workers; 10 Indigenous patients)	Interviews and focus group^d^	8

^a^mHealth: mobile health.

^b^CAT: critical appraisal tool, maximum score is 11.

^c^mHealth interactive: integrated app used for access to health information or personal monitoring of health determinates that allows for information exchange (eg, peers and service providers).

^d^Thematic analysis.

^e^mHealth personal: autonomous app used for access to health information or personal monitoring of health determinates with no interactive capabilities.

^f^Inductive analysis.

^g^Rapid qualitative analysis.

^h^Hybrid approach to qualitative analysis.

^i^Kaupapa Maori approach.

^j^Narrative approach to qualitative analysis.

^k^Yarning approach.

### Thematic Synthesis

#### Overview

Our interpretation of these qualitative findings shows commonalities between Indigenous patients and service providers perceptions of mHealth. We have collectively termed both as “end users” hereafter unless explicitly stated otherwise. Common themes across end users were: the importance of mHealth or digital literacy, mHealth as a facilitator for connection and support, mHealth content that needed to be culturally relevant, and data security and confidentiality. Two themes emerged that were unique to service providers including the importance of mHealth supporting rather than replacing service providers, and the role of workplace champions and organizational capacity for influencing uptake and sustainability of mHealth.

#### mHealth Literacy

Access to the required hardware (mobile or smartphones and touch screen tablets) and systems (network coverage and IT) was identified as an important influence to mHealth uptake by end users. Service providers noted barriers to accessing the mHealth hardware and systems with reasons including regionality and workplace restrictions [40,42,43,45,46]. Service providers held a perception that mobile phones were not prevalent or accessible to patients due to cost [46] and remote location [42]. However, Indigenous patients saw themselves as competent and confident users of technology and mobile phones for everyday life [31,33-38,46,47]. Yet, technology difficulties and lack of device access were still raised in several studies [31,34-36,46,47]. Some studies noted concerns end users had relating to the digital literacy required for mHealth [40,42,44].

Low levels of IT literacy pose a challenge to electronic mental health adoption. Unfamiliarity with different ways of using technologies impedes the utilization of the approach by both service providers and community members. Poor IT literacy within communities was attributed to limited access to technology…

42

Limited confidence in using new technology such as mHealth initiatives was identified as a barrier to uptake for service providers [40,44]. The investment of time and effort into appropriate mHealth training and ongoing support was suggested as a mitigation strategy for technical difficulties for end users [36,40].

Age and generational implications were raised as influential factors to the uptake of mHealth. Whether implicitly or explicitly, end users perceived mHealth would be more applicable and accepted by younger people [31,33,40-42,44,46]. Service providers perceived that older people have limited or no access to mobile phones and thus would have lower digital literacy [40,41,46]. Interestingly, these age-related barriers were not reflected by Indigenous patients in this Australian study.

Most older interviewees did not appear to have any major issues with knowledge on how to access phone features.

36

End users noted the importance of mHealth resources easy to understand and use for a confident user experience. A notable motivation for service providers to use a mHealth resource was for it to be uncomplicated and easy to use [39,40,45-47]. Likewise, Indigenous patients advised that if mHealth platforms were complex, slow, or used too much data, uptake and sustained use was less likely [31,33,35,38,46]. The importance of clear and concise language and the avoidance of jargon in mHealth messaging was noted to encourage comprehension for end users [31,37,38,41,43,47,48]. Service providers stated the importance of mHealth content being appropriate to the learning styles, health knowledge, and communication styles of their patients [39-41,43,44,46,47]. Indigenous patients were enthusiastic about the potential benefits mHealth provided in accessing relevant information for their health journey [33-35,37,38,46-48].

Participants spoke of parents being technologically savvy, and parents referred to accessing apps, YouTube clips, social media and the internet from their mobile phones for infant feeding information and support prior to the Growing healthy program.

47

The incorporation of visual and audio capabilities was suggested by end users to create a better understanding of the content [31,35-41,44,46-48]. mHealth may provide an appropriate tool to bridge health knowledge gaps [42] and enable education and empowerment for health care.

Several interviewees described how the iECG device provided unique opportunities to engage patients in education around AF and their heart, and to empower patients to find out more about their heart health.

45

#### mHealth as a Facilitator for Connection and Support

End users found mHealth an appropriate resource to facilitate engagement, connection, and support within health care systems. Indigenous patients appreciate that mHealth facilitated connection to support people, along with health care providers [31,33-38,47,48]. Likewise, service providers viewed mHealth as important for connection to patients with the added facility to connect with professional colleagues [40-42,46].

mHealth was found to provide a sense of reassurance and encouragement across a range of health journeys for Indigenous patients, including perinatal health [35,47], patients living with mental health challenges [34,37], and people on a smoking cessation journey [36,48]. Indigenous patients suggested mHealth could enable a web-based community to connect with others on similar health journeys [31,33-38,46]. Moreover, Indigenous patients appreciated the capability of mHealth to share health knowledge with family and support people in their lives [33,35,47,48].

Participants valued sharing advice and experience-based information with their families, partners or wider virtual communities, such as Facebook groups.

35

Service providers found that mHealth encouraged trust with patients while creating a collaborative environment with other health staff [40-42,46]. mHealth was found to provide professional peer support [40,46] while streamlining clinical communication and encouraging service provider collaborations [42].

Communication across services working with the same client may help to ensure nonoverlapping of interventions and resources.

42

Australian service providers noted mHealth broke down the barriers of patient engagement, “equalising the power imbalance often present in their relationships with clients” [40]. Service providers attributed this to the app acting as an impartial entity, encouraging person-centered care [40]. First Nations, Inuit, and Métis youth in Canada explained that having a mobile phone would enable them to connect with health professionals as well as on behalf of peers in emergency situations [34]. Youth in Australia found that mHealth provided connection to service providers while adding anonymity and privacy to the navigation of their mental health journey.

Some may have felt known in a small community or simply hesitant to engage a service because they felt uncomfortable. The app allowed them a choice in health care that was previously unavailable.

37

#### mHealth Content Needed to Be Culturally Relevant

End users stated the importance of mHealth including culturally relevant imagery and language to enable engagement, trust, and relatable connection. The inclusion of culturally relevant language and imagery was important for Indigenous patients to encourage engagement and build trust in mHealth content [31-33,37,38,46,47]. Likewise, service providers suggested the need for culturally applicable imagery and language in mHealth content in several studies [39-41,43,44,46,47]. End users suggested the translation of mHealth content to traditional language would enable comprehension of content as well as increase uptake [31,33,40,43,46].

…participants were keen to engage with apps that included Māori language, tikanga and knowledge.

33

Culturally relevant graphics, voices, animation, and optional short video clips may assist in engagement with the content, improve understanding, and overcome literacy issues.

31

Recommended features of a technology resource included a look and feel that was user-friendly, aesthetically pleasing (e.g., more visuals, Indigenous artwork and potentially Indigenous language for more remote communities), easy to read, quick to navigate, and interactive (e.g., notifications, touch screen, user online status shown).

46

Yet, the acknowledgment of diversity in cultural relevance was an important implication noted by Indigenous patients [31,33,38], namely, the tailoring of dialect [31,38] and that content be appropriate to the local cultural peoples [33], to ensure mHealth is not dismissive of cultural diversity.

Findings suggest mHealth can assist in developing cultural competence through gaining a better understanding of cultural diversity, histories, and traditional languages. In Australia, Indigenous patients advised the importance of including cultural determinants such as colonization, intergenerational trauma, and identity within mHealth content [31,38]. In Aotearoa, New Zealand, Māori patients chose to use traditional terminology in the thematic findings of mHealth exploration, acknowledging the importance of the cultural determinants of health [33,35]. mHealth was found to be an important resource to support culturally competent health care delivery for locum service providers in Canada.

Respondents expressed gratitude that the app now exists as an important tool for use in training and orienting new hires to Nunavut’s cultural and language context.

39

#### mHealth Security and Confidentiality

Security and privacy consistently emerged with Indigenous patients across several studies with differing views and implications [31,34,35,37,46]. Povey et al [31] found Indigenous patients were largely dismissive of privacy issues with regard to mHealth, noting that personal information held on phones such as photos, or emails being seen would worry them more. There were, however, concerns raised about the privacy and confidentiality of information being shared during group discussions embedded in mHealth [46]. In addition, Māori women felt a sense of intrusion when using their mobile phone to seek health advice [35]. This intrusion was due to third-party systems, not necessarily a mHealth resource.

…emphasised privacy concerns whereby they encountered personalised advertising on Google and Facebook that was based on previous searches done on the device.

35

Importantly, mHealth offered the opportunity of anonymous support for patients wishing not to engage with health services face to face [31,34,37]. Access to their own phone provided a sense of privacy and a safety net for Indigenous patients in Canada [34]. Likewise, Indigenous patients in Australia appreciated the facility of remote support seeking with the avoidance of unwanted in-person contact.

The ability to interact with the app privately, without anyone else needing to be present, meant that youth who may have been reluctant or afraid to speak to family members or health care professionals in a face-to-face setting could still access support.

37

#### mHealth Supporting Rather Than Replacing Service Providers

Service providers stated the importance of mHealth needing to support established workloads and practices rather than being an onerous addition to established workloads. Service providers raised uncertainties about the sustainability of mHealth, and how their roles and responsibilities may change with the implementation of mHealth [40-42,44,46]. The perceived “lack of fit” with established work practices was a professional barrier identified [40,42,44]. Service providers suggested that mHealth should be considered as a complementary resource in addition to “in person” and physical resources [40,41,46]. Service providers in Australia found mHealth may be more useful for staff lacking experience and confidence in health practice.

Gatekeepers less experienced in suicide prevention may find a resource more useful than more experienced or confident gatekeepers.

46

Service providers saw the benefit of mHealth as an educational tool to develop skills and knowledge. Service providers in Australia liked the health promotion opportunity a smartphone-enabled electrocardiogram (ECG) provided [45]. Service providers in Papua New Guinea valued the guidance capabilities mHealth provided them for clinical malaria treatment procedures [43]. Service providers in Australia identified mHealth as an appropriate resource to gain professional skills and knowledge in interviewing and counseling [40,44]. A smartphone-enabled ECG (ie, iECG) was found to have an indirect educational effect on service providers in Australia.

Some staff also spoke of how using the device for screening led them to want to learn more about AF and cardiovascular disease themselves in their professional role.

45

#### Workplace and Organizational Capacity

Workplace leadership, capacity, and strategic direction emerged as influencing factors to the uptake and sustainability of mHealth for service providers working with Indigenous populations [39,40,42,44,45].

Workplaces that have leaders and champions to drive and support mHealth were a central factor in enabling mHealth uptake. The presence of enthusiastic managers and eager IT champions had a positive effect on the workforce’s interest in mHealth resources with service providers in Australia [42,44]. Workplace leaders that did not perceive the need for or effectiveness of mHealth were often a barrier to the uptake by service providers [42,44]. The advocacy of mHealth from leadership was an influencing factor to acceptance:

Having leaders within the organization showing interest and providing direct support was perceived to facilitate uptake. It created incentives and provided opportunities for service providers to reflect and evaluate the utility of the electronic mental health approach.

42

Workplace staff capacity and retention contributed to the opportunities service providers had to commit to mHealth implementation [40,42,44]. High turnover of staff contributed to a lack of sustained mHealth knowledge and skill within the workplace [40,42,44]. The significance of investment into continued staff training and development was seen as important for mHealth success [40,42,45]. Limited workload capacity due to underresourcing impeded mHealth delivery [40,42,44] and restricted service providers’ capacity to engage in mHealth.

…in many services, demanding workloads left the workers with little or no opportunity to incorporate new skills into their existing work practices…

44

A workplace culture that supports and drives the use of health innovations was shown to positively impact service providers’ perception of mHealth. The absence of health innovation priorities in workplace strategies caused a sense of ambivalence and ineptness toward the need for mHealth among service providers [39,42,44]. Workplaces that invested in systems, valued innovation, and had supportive leadership, positively influenced service providers’ perception and engagement with mHealth tools [40,42,44]. Alignment of the health innovation with organizational principles was found to influence uptake.

Uptake of electronic mental health approaches was dependent upon the perceived fit of the innovation to the organization’s priorities.

42

## Discussion

### Principal Results

This review found that both Indigenous patients and service providers are enthusiastic about the role that mHealth can play in health service delivery.

Common themes across end users were: importance of mHealth or digital literacy, mHealth as a facilitator for connection and support, mHealth content needed to be culturally relevant, and data security and confidentiality are a priority. Two themes emerged that were unique to service providers: the importance of mHealth supporting rather than replacing service providers and the role of workplace champions and organizational capacity for influencing the uptake and sustainability of mHealth.

In this review, most included studies stated the importance of relevant cultural imagery and language, which enabled greater comprehension of mHealth messaging and increased engagement by end users [31-33,37-41,43,44,46,47]. Cultural content needs to account for the heterogeneity of Indigenous peoples, appropriate to location, language, people, and knowledge systems. This creates a challenge for mHealth developers and researchers alike in having 1 product with the capability to be distributed to a culturally diverse audience. Regarding language, Varnfield et al [49] increased their scope of patient engagement with their mHealth app being “available in several different selected languages.” This demonstrates that mHealth has the potential to be adaptive with its content.

Similar to the included study findings of this review, mHealth has been shown to enable patients to engage with their health care providers more effectively as well as connect with peers on similar health care journeys [21]. Moreover, our findings support other reviews reporting health care providers who found mHealth improved communication between their patients and colleagues [15,16].

Our findings showed the importance of workplaces and their leadership in influencing the uptake of mHealth [39,40,42,44,45]. Likewise, Palacholla et al [50] found leadership and organizations that were supportive and facilitated digital health adoption in clinical settings. An important factor when implementing health service innovation is localized agenda setting being led by need, want, and appropriateness [51]. Within a mHealth context, Gagnon et al [52] found health professionals considered their workplace environment as one of the top contributing factors to adoption. Engaging health care organizations as a partner to support mHealth may offer the greatest opportunity for sustained uptake.

Other systematic reviews conducted to understand the influencing factors to mHealth uptake show a strong correlation with the findings presented here. Namely, the principal influencers for adoption are the mHealth design, personal perceptions of mHealth, and the workplace environment [16,21,52], which suggest that co-design may offer an effective methodology for sustained mHealth uptake with Indigenous populations and service providers that work with Indigenous populations.

Early engagement with the Indigenous community within eHealth research and implementation has shown to offer the greatest opportunity for acceptability, and local advocacy [22,23,53,54]. Moreover, this model of prioritizing community partnership and co-design is recommended by governing ethical guidelines on research with Indigenous peoples internationally to achieve beneficial research outcomes [55-58]. Despite this, Eyles et al [59] found a lack of co-design methods for minority and Indigenous groups internationally in the development of mHealth interventions. With the novelty of mHealth along with the cultural considerations involved in the study population, it would be practical to enter a colearning and cocreation relationship to achieve mutually beneficial outcomes.

In conclusion, there has been considerable growth in qualitative research exploring contextual factors in relation to mHealth uptake in non-Indigenous populations, yet less so for Indigenous populations. To our knowledge, this is the first review of qualitative studies that provides an understanding of the influential factors for both patients and service providers for Indigenous populations in relation to mHealth.

### Strengths, Limitations, and Future Directions

Having 2 reviewers from diverse cultural backgrounds and gender orientations independently screening improved the quality of this meta-synthesis. The authors are a multidisciplinary team with a breadth of expertise in this review focus (psychology, digital health, qualitative research, and Indigenous health). Using a meta-aggregative approach to analyze the findings ensured cultural learnings identified by researchers’ conducting the original studies were not lost by the reviewers. The quality appraisal tool used a modified version of the CASP qualitative checklist, with the additional question locating the researchers’ cultural or theoretical standpoint, improving the cultural rigor of this critical appraisal tool. Most studies were of medium to high quality, and the quality appraisal tool can be found in Multimedia Appendix 2.

Our review has some limitations; first, the searches were restricted to peer-reviewed literature published in 5 databases (PubMed, CINAHL, Embase, PsycINFO, and Web of Science). Second, publication bias may have occurred due to the subjective quantifying of studies reporting on one or more outcomes via qualitative research methods. Finally, the results of this study are based on the meta-synthesis of qualitative data, which is inherently subjective. There are studies included from all countries (except the Sápmi region), but there are still only a few studies in each country, and so more work is needed. Papers need to report not only on patients’ perspective but other end users to gain a full understanding of the perceptions of mHealth in supporting health care with Indigenous populations.

### Conclusions

This review used meta-aggregation to summarize the findings of 17 qualitative studies on the experiences and perceptions of mHealth with Indigenous populations and the service providers that work with Indigenous populations. mHealth end users are enthusiastic about the role that mHealth can play in Indigenous health service delivery. There is a need for mHealth design to center end users within a co-designed approach with Indigenous people. There is recent work driving this agenda in an Australian context [60]. Allowing end users to suggest localized agenda setting through co-design may provide an opportunity for ownership, championship, and mitigation of barriers in mHealth implementation. Future research should partner with key representatives (eg, patients, health care professionals, and executive leaders) in the mHealth design appropriate to the purpose, people, setting, and delivery.

## Appendix

Multimedia Appendix 1 Qualitative systematic review search string example.

Multimedia Appendix 2 CASP tool with additional JBI question.

## Abbreviations

CASP: Critical Appraisal Skills Programme
ECG: electrocardiogramJBI: Joanna Briggs Institute
mHealth: mobile health
PROSPERO: International Prospective Register of Systematic Reviews

## Data Availability

The search strategy is available in Multimedia Appendix 1. Any additional data are available upon request from the corresponding author.
