# Supplementary figures and images for: Influencing Factors to mHealth Uptake With Indigenous Populations: Qualitative Systematic Review
Source: JMIR Mhealth Uhealth. 2023 Jun 23;11:e45162. doi: 10.2196/45162 (PMC10337452; doi:10.2196/45162)

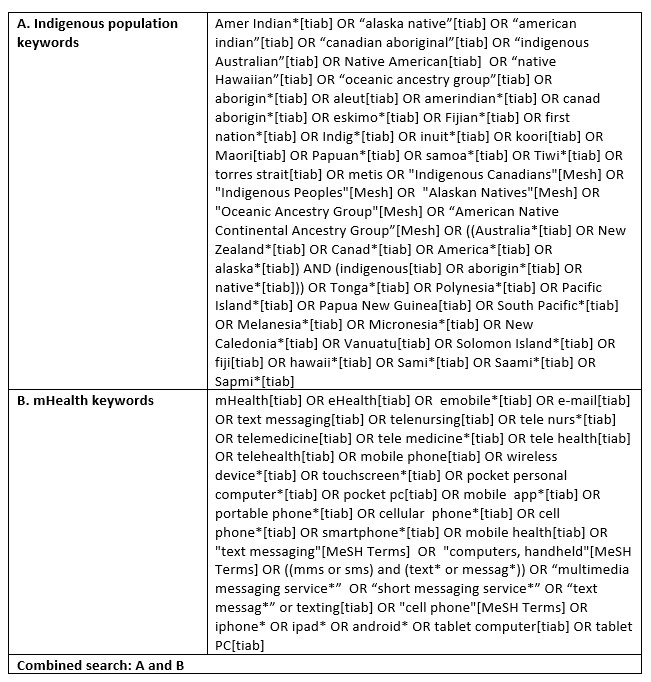

Supplement: Multimedia Appendix 1 [file mhealth_v11i1e45162_app1.png]
