# Supplementary material for: Influencing Factors to mHealth Uptake With Indigenous Populations: Qualitative Systematic Review
Source: JMIR Mhealth Uhealth. 2023 Jun 23;11:e45162. doi: 10.2196/45162 (PMC10337452; doi:10.2196/45162)
Supplement: Multimedia Appendix 2 [file mhealth_v11i1e45162_app2.pdf]

Items assessed	Number of studies: 17		
	Yes	No	Can't tell
1. Was there a clear statement of the aims of the research?	15	2	0
2. Is a qualitative methodology appropriate?	16	0	1
3. Was the research design appropriate to address the aims of the research?	15	0	2
4. Was the recruitment strategy appropriate to the aims of the research?	16	0	1
5. Was the data collected in a way that addressed the research issue?	13	1	3
6. Has the relationship between researcher and participants been adequately considered?	8	6	3
7. Have ethical issues been taken into consideration?	14	3	0
8. Was the data analysis sufficiently rigorous?	11	3	3
9. Is there a clear statement of findings?	17	0	0
10. How valuable is the research?	17	0	0
11. Is there a statement locating the researcher culturally or theoretically?	6	7	4
